# Acoustic Hyper-Reactivity and Negatively Skewed Locomotor Activity in Children With Autism Spectrum Disorders: An Exploratory Study

**DOI:** 10.3389/fpsyt.2018.00355

**Published:** 2018-08-06

**Authors:** Hidetoshi Takahashi, Toru Nakamura, Jinhyuk Kim, Hiroe Kikuchi, Takayuki Nakahachi, Makoto Ishitobi, Ken Ebishima, Kazuhiro Yoshiuchi, Tetsuya Ando, Andrew Stickley, Yoshiharu Yamamoto, Yoko Kamio

**Affiliations:** ^1^Department of Child and Adolescent Mental Health, National Institute of Mental Health, National Center of Neurology and Psychiatry, Tokyo, Japan; ^2^Department of Advanced Neuroimaging, Integrative Brain Imaging Centerm, National Center of Neurology and Psychiatry, Tokyo, Japan; ^3^Graduate School of Education, The University of Tokyo, 2Tokyo, Japan; ^4^Department of Psychosomatic Research, National Institute of Mental Health, National Center of Neurology and Psychiatry, Tokyo, Japan; ^5^Department of Stress Sciences and Psychosomatic Medicine, Graduate School of Medicine, University of Tokyo, Tokyo, Japan; ^6^Stockholm Center for Health and Social Change, Södertörn University, Huddinge, Sweden

**Keywords:** acoustic hyper-reactivity, acoustic startle reflex, autism spectrum disorders, endophenotypes, locomotor activity, prepulse inhibition, sensorimotor gating

## Abstract

Investigation of objective and quantitative behavioral phenotypes along with neurobiological endophenotypes might lead to increased knowledge of the mechanisms that underlie autism spectrum disorders (ASD). Here, we investigated the association between locomotor dynamics and characteristics of the acoustic startle response (ASR) and its modulation in ASD (*n* = 14) and typically developing (TD, *n* = 13) children. The ASR was recorded in response to acoustic stimuli in increments of 10 dB (65–105 dB SPL). We calculated the average ASR magnitude for each stimulus intensity and peak-ASR latency. Locomotor activity was continuously measured with a watch-type actigraph. We examined statistics of locomotor activity, such as mean activity levels and the skewness of activity. Children with ASD had a significantly greater ASR magnitude in response to a weak acoustic stimulus, which reflects acoustic hyper-reactivity. The skewness of all-day activity was significantly more negative in children with ASD than those with TD. Skewness of daytime activity was also more negative, although only of borderline statistical significance. For all children, the higher mean and more negatively skewed daytime activity, reflecting hyperactivity that was associated with sporadic large daytime “troughs,” was significantly correlated with acoustic hyper-reactivity. The more negatively skewed locomotor activity occurring in the daytime was also associated with impaired sensorimotor gating, examined as prepulse inhibition at a prepulse intensity of 70 dB. This comprehensive investigation of locomotor dynamics and the ASR extends our understanding of the neurophysiology that underlies ASD.

## Introduction

Expectations of translational research in relation to being able to determine the biological pathology and fully effective treatments for autism spectrum disorder (ASD), are increasing. Specifically, the expectation of acquiring a deeper understanding of objective and quantitative behavioral and neurobiological indices is growing. Such indices will contribute to the progress of basic and clinical research and lead to the identification of promising ASD phenotypes or endophenotypes.

One promising objective and quantitative endophenotype for translational research is the acoustic startle response (ASR) and the way it is modulated, including aspects such as habituation and prepulse inhibition (PPI). The neurophysiological indices of ASR are often used to assess information processing differences across (ethnic) groups and species as they can be evaluated by using similar nonverbal experimental designs (1, 2). Sensory abnormalities often occur in people that have ASD (3, 4) and are regarded as important elements in this disorder. Among the ASR indices, an increased ASR magnitude to weak stimuli might act as a useful indicator for translational research, especially when considering acoustic hyper-reactivity. For example, recent research (5, 6) has indicated that in response to weak stimuli, peak-ASR latency is prolonged and ASR magnitude is greater in ASD children when compared to those with typical development (TD). Importantly, the difference in these indices which were associated with emotional/behavioral difficulties in ASD children (6), exhibited a fair to moderate degree of stability over a follow-up period of 1 year (7).

Another promising candidate index for translational research is locomotor dynamics. Locomotor activity is a behavioral index that has been examined in both basic (animal) and clinical research in relation to psychiatric and developmental disorders (8–12). In terms of ASD, locomotor activity is frequently examined using an actigraph, primarily to document atypical sleep patterns (13–17). However, statistical measures of daytime, waking locomotor activity have not been well established in ASD. This is an important oversight, especially as recent studies of depression (18, 19) and attention-deficit hyperactivity disorder (20–22) have highlighted the usefulness of measures such as mean locomotor activity during waking periods. Compared with the ASR, measuring locomotor activity with an actigraph or by video-recording can be less invasive and more continuous, even during infancy (23). Thus, identifying the clinical significance of locomotor activity during early development might help uncover fundamental mechanisms in the psychopathology of ASD.

Thus, this study's aim was to examine the association between locomotor activity and ASR indices in children with ASD. Locomotor activity was recorded by actigraph and analyzed. We examined several ASR properties, including the magnitude of the ASR to sounds of varying intensities, peak-ASR latency, habituation, and PPI. Our hypothesis was that acoustic hyper-reactivity (a greater ASR magnitude to weak stimuli)—which is related to ASD—would also be related to the dynamic properties of locomotor activity measured in daily life.

## Materials and methods

### Participants

Fourteen Japanese children with ASD (13 boys) and 13 with TD (10 boys) participated in the study (age: 7–16 years). Participants were recruited through locally placed advertisements. Experienced child psychiatrists assigned diagnoses after reviewing the children's medical records and performing a clinical interview based on the Diagnostic and Statistical Manual of Mental Disorders, Fourth Edition, Text Revision (24). The Autism Diagnostic Interview-Revised (25) and the Autism Diagnostic Observation Schedule (26) were used to confirm diagnoses. Neither sex, age (age in months; ASD 125.6 ± 30.9; TD 138.5 ± 38.2; *U* = 76, *p* = 0.467), or the estimated intelligence quotient (IQ: ASD 105.7 ± 23.3; TD 104.7 ± 18.3; *U* = 31, *p* = 0.958) differed significantly between the two groups. Additionally, when using the Wechsler Intelligence Scale for Children-Third Revision (27) the estimated IQ of every child in the study was above 70. None of the children were smokers or were currently being medicated with psychotropic substances. In addition, none of them had any degree of hearing loss according to the results from the annual school health check-up which includes bilateral hearing screening of 1,000 Hz acoustic stimuli larger than 30 dB and 4,000 Hz acoustic stimuli larger than 40 dB. Further, none of the children had any abnormalities of the central nervous system apart from autism. Exclusion criteria for the TD group included having a previous or current psychiatric diagnosis or learning disability.

### Ethical approval and informed consent

This study was undertaken in accordance with the principles laid out in the 1964 Helsinki Declaration and its subsequent amendments and institutional review-board approval was granted by the Research Ethics Committee of the National Center of Neurology and Psychiatry (#A2013-112) and the research ethics committee of the Graduate School of Education, the University of Tokyo (#13-119). Before being accepted into the study, the study procedures were explained in detail and written informed consent was obtained from each participant and their parents.

### Startle response

Details of the stimulus-presentation and eyeblink-acquisition methodology have been presented previously (5, 6, 28, 29). The startle paradigm that was used to test participants comprised three blocks, and we examined the following ASR measures: (i) average eyeblink magnitude in response to each of the five pulse intensities (65, 75, 85, 95, and 105 dB SPL) in block 1; (ii) average peak-ASR latency; (iii) ASR habituation during each test period, calculated as the reduction in the ASR magnitude percentage (at 105 dB SPL) between the first and third blocks; (iv) PPI at prepulse intensities of 65, 70, and 75 dB SPL. Regarding prepulse intensity, each PPI was calculated as the percentage reduction in the ASR magnitude in block 2 between the pulse alone and the pulse with prepulse trials. As the brain mechanisms subserving PPI do not become sufficiently mature until children become 8–10 years old (30–32), we did not examine ASR measures in four boys (two with ASD and two with TD) who were less than eight years old. In addition, because one boy with ASD could not tolerate the startle stimulus, he did not finish the session and his data were therefore not used in the subsequent statistical analysis.

### Assessment of locomotor dynamics

All participants were instructed to wear the MicroMini Motionlogger actigraph (Ambulatory Monitors Inc., Ardsley, NY, USA) (33) on the wrist of their non-dominant hand for more than seven days during school vacations in spring, summer, or winter (TD: 7.7 ± 1.9 days, ASD: 7.8 ± 1.8 days, U = 80, *p* = 0.574). Children were expected to wear this device throughout the study period, except for when bathing, or during rigorous exercise, or when undertaking any other activity that might cause damage to the device, and to lead their lives normally during the period of actigraphic recordings.

This particular actigraph has been used extensively in clinical research (8, 33–35). Locomotor activity was assessed with a uni-axial piezo-electronic accelerometer sensor that is able to detect even minor differences in body acceleration (≥0.01 G/rad/s). In the current study, we used zero-crossing counts that were collected together for each 1-min epoch as a measure of locomotor activity. After children returned the actigraph, ActMe software (ver. 3.10.0.3, Ambulatory Monitoring Inc., Ardsley, NY, USA) was used to download their activity data. Any locomotor activity data that were collected when participants were not actually wearing the device were not included in the analysis. This was done using Action W-2 software (ver. 2.4.20, Ambulatory Monitoring Inc., Ardsley, NY, USA) to manually label the bad time periods (bins) when the participants had taken off the device.

Sleep–wake cycles were scored and sleep measures were also analyzed using Action W-2 software. Sleep epochs were determined based on the Cole-Kripke algorithm (36). One boy with ASD and two TD girls and two TD boys were excluded from the actigraph behavioral data analysis because they did not wear the actigraph for a sufficient period of time during the daytime.

Sleep measures obtained through actigraphy were (a) sleep duration: the total sleep time in minutes between the time of sleep-onset and waking time; (b) sleep latency: the total time in minutes between going to bed and sleep onset; (c) wake-after-sleep onset (WASO): the number of minutes spent awake during the night after the onset of sleep; and (d) sleep efficiency: the ratio between the total sleep time and amount of time spent in bed at night. Actigraphic sleep parameters were calculated each night for each participant and then averaged for each participant.

We also calculated the mean, standard deviation, skewness, and kurtosis of locomotor activity as these can demonstrate behavioral alterations associated with psychiatric disorders or psychological states (10, 19). For example, higher or lower than normal mean activity levels can characterize states related to psychomotor retardation or agitation, respectively. A right-skewed distribution indicates the presence of extreme values higher than their mean (“occasional bursts”). Right-skewed activity levels with a low mean can characterize the increased intermittent patterns of locomotor activity that are exhibited by patients with major depressive disorder (19). Further, this pattern of locomotor activity has been significantly associated with the worsening of depressive mood in healthy adults and depressive patients (10, 19). Therefore, we evaluated the association between these statistics and the ASR indices. As no significant differences between groups were detected in the standard deviation or kurtosis of locomotor activity, detailed results for these statistics are not reported in the text.

### Statistical analysis

Chi-square tests (and when necessary Fisher's exact tests) were used to examine categorical aspects of the participants' demographic data. As most of the variables relating to ASR and locomotor activity were not normally distributed, nonparametric analyses were performed. Specifically, differences in mean parameter values were examined with the Mann-Whitney *U*-test. Associations between the variables were computed with Spearman's rank order correlation coefficients. As some children were excluded from the ASR and/or actigraph behavioral data analysis, data from 10 children (1 girl) with ASD and 8 children (1 girl) with TD were included in the analysis of the relationship between ASR measures and locomotor activity statistics, while data from 11 children (1 girl) with ASD and 11 (3 girls) with TD were included in the analysis of the relationship between ASR measures and sleep measures. A Bonferroni adjustment for multiple comparisons was used to correct significance levels. Statistical significance was set at *p* < 0.05. All analyses were conducted with SPSS Version 22 (IBM Japan, Tokyo, Japan).

## Results

### Startle measure differences between ASD and control children

Startle measures are presented in Table 1. Children with ASD had significantly prolonged peak-ASR latencies. Additionally, their ASR magnitude was also significantly greater at the 65-, 75-, and 95-dB stimulus intensities. A trend toward a greater ASR magnitude was also observed in ASD children at the 85-dB stimulus intensity. For PPI, the only significant difference between groups was for the prepulse intensity of 65-dB. Statistically significant differences in habituation or PPI were not observed between the groups at any of the other prepulse intensities.

**Table 1:** Acoustic startle response measures.

	**Typical development**		**Autism spectrum disorders**	
	**Mean**	***SD***	**Mean**	***SD***	***U***	***p***
Peak Startle Latency (ms)	66.0	11.7	90.4	17.3	19	0.006
**ACOUSTIC STARTLE RESPONSE (MICROVOLTS)**						
65 dB	33.6	20.9	53.6	27.1	25	0.020
75 dB	27.8	11.6	47.9	25.0	28	0.033
85 dB	32.6	7.7	61.4	46.6	31	0.052
95 dB	38.1	18.5	66.5	57.5	23	0.014
105 dB	55.2	37.3	85.6	71.4	43	0.250
Habituation (%)	29.3	7.3	22.4	18.7	24	0.248
**PREPULSE INHIBITION (%)**						
65 dB prepulse	35.6	20.2	14.1	13.4	17.5	0.008
70 dB prepulse	32.1	19.8	22.0	19.4	40	0.290
75 dB prepulse	33.0	19.3	33.6	21.5	59	0.922

*SD, Standard deviation; Mann-Whitney U-test. Number of participants (Typical Development: Autism Spectrum Disorders) for Peak startle latency = 11:11; Acoustic startle magnitude (65 dB) = 11:11; (75 dB) = 11:11; (85 dB) = 11:11; (95 dB) = 11:11; (105 dB) = 11:11; (Habituation) = 9:8; Prepulse inhibition (65-dB prepulse) 10:11; (70-dB prepulse) 10:11; (75-dB prepulse) 11:11*.

### Differences in locomotor dynamics between children with autism spectrum disorders and controls

Locomotor activity is presented in Table 2. Significantly more negative skewness—defined as a left-skewed distribution (or a long left tail relative to the right tail) with extreme values lower than their mean—was observed for all-day activity in children with ASD, indicating an increase in sporadic large “troughs” below mean activity levels. The skewness of daytime activity was also more negative in those with ASD, although it was only of borderline statistical significance. No other significant differences in locomotor activity or any differences in sleep measures were observed between the groups.

**Table 2:** Locomotor dynamics.

	**Typical development**		**Autism spectrum disorders**	
	**Mean**	***SD***	**Mean**	***SD***	***U***	***p***
**SLEEP MEASURES**						
Sleep duration (minutes)	517.7	64.2	516.2	39.4	87	0.846
Sleep latency (minutes)	7.8	3.6	7.9	3.3	87	0.846
Wake after sleep onset (minutes)	28.8	20.5	32.4	24.5	84	0.734
Sleep efficiency (%)	94.5	3.9	93.8	4.6	87	0.846
**LOCOMOTOR ACTIVITY STATISTICS ALL DAY ACTIVITY**						
Mean	147.9	18.9	159.0	7.5	35	0.117
Skewness	0.0	0.2	−0.2	0.2	28	0.042
**DAYTIME ACTIVITY**						
Mean	231.7	32.0	239.5	18.4	41	0.243
Skewness	−1.1	0.6	−1.6	0.5	31	0.066

*SD, Standard deviation; Mann-Whitney U test. Number of participants (Typical Development: Autism Spectrum Disorders) for Sleep measures = 13:14; Locomotor activity statistics = 9:13*.

### Relationship between locomotor dynamics and startle measures

When all the children were combined, mean activity levels for daytime activity were significantly correlated with ASR magnitude for the 75-dB stimulus (rho = 0.484, *p* = 0.042). Likewise, activity-skewness values for daytime activity were significantly correlated with ASR magnitude for the 65-dB stimulus (rho = −0.626, *p* = 0.005), 85-dB stimulus (rho = −0.499, *p* = 0.035), and PPI at 70-dB prepulse intensity (rho = 0.566, *p* = 0.018). The negative skewness of daytime activity was significantly correlated with the ASR magnitude to a 65-dB stimulus even after a Bonferroni correction for multiple comparisons was applied. No other significant correlations were observed between locomotor activity or any sleep parameter and the ASR measures.

These relations were also confirmed within each group because ASR magnitude for the 65-, 75-, and 85-dB stimuli was either significantly different or tended to differ between diagnoses. This analysis revealed a statistically significant correlation between daytime skewness and ASR magnitude for the 65-dB stimulus (rho = 0.745, *p* = 0.013) in the ASD group.

## Discussion

This study investigated locomotor activity that was measured by actigraph in ASD and TD children. We also investigated the ASR, its modulation by PPI and habituation, and how these indices were related to locomotor dynamics. Results indicated that locomotor activity skewness for children with ASD was significantly more negative for all-day activity, and tended to be more negative for daytime activity. When all children were combined, the mean and skewness values for daytime locomotor activity correlated with several ASR measures, including ASR magnitude for 85 dB stimuli or weaker and PPI at a 70-dB prepulse intensity. Additionally, in the ASD group, as the ASR magnitude to a weak 65-dB stimulus increased, the skewness values for daytime activity became more negative. Our results thus suggest that atypical hyperactivity behavior observed in locomotor dynamics might be caused, in part, by acoustic hyper-reactivity to weak acoustic stimuli.

To our knowledge, this study is the first to report a relation between locomotor dynamics and ASR indices in humans. The skewness of all-day locomotor activity was more negative in ASD than in TD children. These results suggest that negatively skewed all-day activity might serve as a promising quantitative behavioral index related to ASD.

Higher mean activity levels and more negatively skewed values for daytime locomotor activity characterize behavior in children as being hyperactive with sporadic large “troughs” in daytime activity. In the current study, these values were significantly linked to an increased ASR magnitude to weak 65-dB stimuli, a characteristic which has been associated with several autistic traits in ASD and TD children (5, 6). Our finding that the daytime skewness in locomotor activity tended to be more negative in those with ASD is consistent with the idea that the prevalence of attention-deficit hyperactivity disorder in ASD is high (37). However, hyperactivity/inattention might be associated with acoustic hyper-reactivity. Thus, the analysis of daytime locomotor activity, especially daytime skewness, might provide promising behavioral phenotypes that are related to clinical features in ASD, such as acoustic hyper-reactivity or hyperactivity/inattention.

Our results support the utility of focusing on third-order statistical moments such as skewness in addition to standard descriptive statistics when characterizing behavioral alterations in ASD children. In a recent study, children with ASD were found to be more active during rest periods than healthy children, although this difference was non-significant in statistical terms. However, in rest periods the kurtosis and skewness of their activity distributions were significantly smaller than those of healthy children (38). Other recent research (18, 19) that investigated the relationship between locomotor dynamics and depressive mood reported that the worsening of depressive mood was linked to a greater intermittency of locomotor activity, as seen in lower mean scores and increased positive skewness values. As higher order statistics successfully capture intermittency or non-Gaussian distributions in natural phenomena, these types of analyses should be useful in assessing the locomotor dynamics of children with ASD. Thus, further investigation of daytime and sleep activity using these higher order statistics might reveal more apparent characteristic behavioral alterations in ASD and other psychiatric disorders.

In this study, ASR measures were related to several aspects of locomotor dynamics, which suggests that basic and clinical research using ASR measures and locomotor dynamics might facilitate a better understanding of the association between ASD and co-occurring psychiatric or developmental conditions. For example, numerous animal studies (although not evaluating skewness of locomotor activity) have reported a relationship between increased locomotor activity and decreased PPI in connection with dysfunction in dopaminergic, serotonergic, and glutamatergic neurotransmitter regulation (39–43). These associations are thought to be related to hippocampal function (40, 43), which suggests an underlying shared biological mechanism between some aspects of locomotor activity and the ASR. We found that for the daytime activity of both groups combined, more negative skewness was significantly associated with smaller PPI at the prepulse intensity of 70 dB, which has been related to some subcategories of autistic traits and with emotional/behavioral difficulties in children with ASD and TD in previous studies (5, 6). In the current study, the skewness of all-day locomotor activity was significantly more negative, and the skewness of daytime locomotor activity more negative (although only of borderline significance) in ASD children, suggesting that a comprehensive investigation of locomotor activity and its relationship with ASR modulation might help clarify the neurophysiological basis of ASD and other clinical problems in children. Additionally, assessing locomotor activity, especially skewness, in daily settings might also serve as a preliminary test to predict ASR indices, including PPI. As locomotor activity can be examined less invasively and more continuously than the ASR even in infancy (23), its evaluation during early development in relation to ASD symptom severity might provide an insight into the fundamental mechanisms that contribute to the broad vulnerability to developmental psychopathology seen in ASD.

The fact that both the ASD and TD groups contained few participants is a major limitation of this study. Even though we were able to identify significantly more negative skewness in the all-day locomotor activity in those with ASD, and significant associations between some aspects of locomotor dynamics and ASR indices, the sample size might nevertheless have been too small to detect other significant differences or associations. For example, the significant relationship between PPI and mean locomotor activity, which has been reported in previous animal studies (39–43), was not observed in this study. In particular, no significant differences were found for the sleep measures despite reports of such differences in previous studies (13–17). This might be related to the small number of children included in this study. Similar sleep problems are experienced at a markedly higher prevalence in school-aged children with ASD (44–83%) than in those with TD, and, atypical sleep patterns, such as prolonged sleep latency (14–17), lower sleep efficacy (14, 16, 17), and longer WASO (13, 14) are frequently reported in children with ASD. However, a previous study (44) reported that although school-age children with Asperger syndrome or high-functioning autism had longer sleep latency and lower sleep efficiency on school days, these differences were not found over the weekend (44), suggesting that sleep patterns in ASD might differ according to the level of daytime activity, and that children with ASD might have difficulty in regulating their school-life rhythm. Thus, the fact that no significant differences were observed in the sleep measures between the ASD and TD groups in this study might be partly due to the locomotor data acquisition period, which was during long seasonal school vacations when participants did not have to adjust to the rhythms of school life. Participants with ASD might exhibit more sleep problems during school days. Future studies with larger samples that include data from both seasonal school vacations and school days are necessary to clarify the relationship of sleep measures to ASR measures.

Additionally, this study only included ASD children without intellectual disabilities and IQ-matched controls, who were mainly boys, while the age span, which might be important for differences in hormone levels, was rather large. By using intellectual disabilities as an exclusion criterion, we aimed to avoid the high rates of participant rejection reported previously (45). However, it is possible that the ASR profile of ASD children with intellectual disabilities might also differ. Moreover, although not much is known about gender differences in the locomotor dynamics of children, sexual hormones are known to have an effect on ASR modulation, such as PPI (1, 2). Thus, future research needs to use larger samples to examine these associations in participants with intellectual disabilities and in both sexes while ensuring that there is a narrower age range.

## Conclusion

The results from the current study suggest that negatively skewed all-day locomotor activity might serve as a promising quantitative behavioral index related to ASD. For all children, acoustic hyper-reactivity (assessed as a greater ASR magnitude in response to weak stimuli) was related to higher levels of locomotor activity and a negatively skewed activity distribution (which reflected hyperactivity that was characterized by large sporadic “troughs,” during the daytime). The more negatively skewed daytime locomotor activity was also associated with impaired sensorimotor gating (i.e., PPI). This comprehensive investigation of locomotor dynamics and the ASR thus extends our understanding of the neurophysiology that underlies ASD.

## Author contributions

HT, ToN, JK, HK, KY, TA, YY, and YK conceived and designed the experiments. HT, ToN, YY, and YK supervised the project. HT and YK confirmed diagnoses. HT, ToN, JK, and TaN performed the experiments. HT, ToN, JK, YY, and YK analyzed the data. HT, ToN, JK, MI, KE, AS, YY, and YK wrote the manuscript. All authors read and approved the final manuscript.

### Conflict of interest statement

The authors declare that the research was conducted in the absence of any commercial or financial relationships that could be construed as a potential conflict of interest.
